# An Ontology-Based Vehicle Behavior Prediction Method Incorporating Vehicle Light Signal Detection

**DOI:** 10.3390/s24196459

**Published:** 2024-10-06

**Authors:** Xiaolong Xu, Xiaolin Shi, Yun Chen, Xu Wu

**Affiliations:** 1 College of Mechanical Engineering and Automation, Liaoning University of Technology, Jinzhou 121001, China; 2 College of Mechatronic Engineering, North Minzu University, Yinchuan 750021, China

**Keywords:** vehicle behavior prediction, deep learning, brake light detection, ontology reasoning

## Abstract

Although deep learning techniques have potential in vehicle behavior prediction, it is difficult to integrate traffic rules and environmental information. Moreover, its black-box nature leads to an opaque and difficult-to-interpret prediction process, limiting its acceptance in practical applications. In contrast, ontology reasoning, which can utilize human domain knowledge and mimic human reasoning, can provide reliable explanations for the speculative results. To address the limitations of the above deep learning methods in the field of vehicle behavior prediction, this paper proposes a front vehicle behavior prediction method that combines deep learning techniques with ontology reasoning. Specifically, YOLOv5s is first selected as the base model for recognizing the brake light status of vehicles. In order to further enhance the performance of the model in complex scenes and small target recognition, the Convolutional Block Attention Module (CBAM) is introduced. In addition, so as to balance the feature information of different scales more efficiently, a weighted bi-directional feature pyramid network (BIFPN) is introduced to replace the original PANet structure in YOLOv5s. Next, using a four-lane intersection as an application scenario, multiple factors affecting vehicle behavior are analyzed. Based on these factors, an ontology model for predicting front vehicle behavior is constructed. Finally, for the purpose of validating the effectiveness of the proposed method, we make our own brake light detection dataset. The accuracy and mAP@0.5 of the improved model on the self-made dataset are 3.9% and 2.5% higher than that of the original model, respectively. Afterwards, representative validation scenarios were selected for inference experiments. The ontology model created in this paper accurately reasoned out the behavior that the target vehicle would slow down until stopping and turning left. The reasonableness and practicality of the front vehicle behavior prediction method constructed in this paper are verified.

## 1. Introduction

Vehicle behavior prediction can effectively improve the ability of self-vehicles to predict the future movements of other traffic participants and enhance the ability to perceive potential risks in dynamic and complex traffic environments. It is the basis for realizing autonomous decision-making and planning of intelligent vehicles, as well as an indispensable perceptual prediction capability in the development process of vehicle automation and intelligence [1,2]. With the deep integration of the automotive industry with information technology and artificial intelligence technology, autonomous driving technology continues to break through, but vehicle behavior prediction still faces many challenges [3]. The interdependence between vehicles, the complex and changing road environment, and the constraints of traffic rules, as well as the uncertainty of the future behavior of vehicles due to the subjective operation of drivers, all put forward higher requirements on the accuracy and robustness of prediction algorithms [4,5]. Therefore, the vehicle behavior prediction module needs to comprehensively consider the movement trends of surrounding vehicles, road conditions, traffic rules, and even weather factors in order to comprehensively capture a wide range of possible future behaviors of vehicles and ensure that accurate and timely decisions can be made in complex traffic scenarios.

Traditional vehicle behavior prediction methods, such as prediction algorithms based on physical models and prediction algorithms based on traditional machine learning models [6,7], are mainly suitable for short-term, stable, and relatively simple driving scenarios due to insufficient performance. In recent years, deep-learning-based methods have become popular due to their good performance in more complex environments compared to traditional methods. The main highlight of deep learning techniques is the ability to implicitly extract features needed to predict vehicle behavior, and some models even take into account vehicle interactions, such as Recurrent Neural Networks (RNN) and Graph Convolutional Neural Networks (GCNN) [8]. However, factors such as environmental conditions and traffic rules still cannot be directly integrated into the prediction model. Moreover, due to the black-box nature of deep learning, the interpretability and reliability of its prediction results are still weak, and this black-box model can easily lead to passengers’ distrust of the machine [9,10]. In contrast, by constructing a domain knowledge base of vehicle behavior, the ontology is able to explicitly express the logical relationship between vehicle states, traffic rules, environmental factors, etc., making the prediction results more comprehensible and traceable. However, it is slightly insufficient in terms of dynamic knowledge updating and feature extraction capabilities. It can be seen that the advantages and disadvantages of the two are complementary to a certain extent. The combination of deep learning and ontology reasoning can fully utilize the advantages of both approaches.

Based on the above analysis, this paper proposes a prediction method for front-end vehicle behavior that combines deep learning techniques and ontological reasoning. It uses deep learning techniques to obtain information about the brake lights of the front vehicle, uses ontology models to integrate multiple factors affecting vehicle behavior, and uses ontological reasoning to make the inferred results interpretable and trustworthy. Specifically, we constructed a front brake light information detection module based on the YOLOv5s model. By introducing the Convolutional Block Attention Module (CBAM) and weighted Bi-directional Feature Pyramid Network (BIFPN), it aims to improve the detection performance of this model in different scenarios. Second, in order to integrate the factors affecting vehicle behavior, we consider the vehicle type, traffic signals, road signs, and car light language information. After that, we define the key entities, attributes, and their interrelationships in the car driving scenario to build an ontology model that can accurately reflect the actual driving environment and has extensive explanatory power in terms of vehicle behavior. Finally, we collect data using a device such as a car recorder to produce a vehicle traveling dataset containing brake light states and use it to train the improved YOLOv5s model. In order to verify the effectiveness of the described method, representative verification scenarios are selected for inference experiments. The experimental results show that the method can not only accurately predict the driving behavior of the front vehicle, but also improve the interpretability and reliability of the prediction results.

The rest of the paper is organized as follows: Section 2 describes the related studies. Section 3 provides an overview of the proposed method. Section 4 describes the related work on improving the detection model. Section 5 details the constructed ontology model. Section 6 analyzes and discusses the experimental results. Finally, Section 7 summarizes the work of this paper.

## 2. Related Work

In this section of our review, we survey the related research endeavors in the domains of automotive light signal detection and vehicle behavior prediction.

In the field of car light inspection, most of the research focuses on the detection and recognition of tail lights in view of their key role in enhancing the road safety and decision-making capability of automated driving systems. To provide a clearer picture of the development path in this area, we systematically classified its principal approaches into two distinct categories: multi-stage processing methods and deep learning methods. The multi-stage processing method is mostly based on the detection of the vehicle or tail target. The first step is to detect the vehicle or rear target in the original image sequence, to extract tail light features based on the detected target, and to judge the state of its tail light. Wang, Z. et al. [11] utilized a convolutional neural network to detect and extract the vehicle contours while specifically extracting the tail light region in the HSV (Hue-Saturation-Value) color space. Furthermore, the accurate detection of tail light pairs is achieved by analyzing and exploiting the correlation between color and position. Chen et al. [12] used a Nakagami image-based approach to localize regions containing headlights for a nighttime driving scenario. A region suggestion network based on a Convolutional Neural Network (CNN) feature map is utilized to generate a set of vehicle object suggestions, combining the two to generate a region of interest (ROI). In the tracking module, a perceptual hashing algorithm is proposed. During the tracking process, the steering signals are detected by analyzing the continuous intensity variations of the vehicle box sequences. Chen et al. [13] proposed a brake light detection system that used the Lab color space to generate a tail light interest region and used the symmetry of tail lights for tail light verification and pairing. Cao et al. [14] localized tail lights based on their shape and color, and established a tail light recognition mechanism based on red-green-blue (RGB) and cyan-magenta-yellow (CMY) color spaces. The method of deep learning utilizes target detection models to directly locate the regions of tail lights, or alternatively, to directly detect the signals of illuminated tail lights. Li, Q. et al. [15] proposed the improved target detection of the YOLOv3-tiny-based algorithm. Firstly, the feature fusion scale is added to the model, and then the Spatial Pyramid Module (SPP) is brought in to extract the multi-scale features; lastly, the focusing loss is introduced to settle the category unbalance issue, and the focusing loss is introduced to realize the accurate recognition of the tail light. Zhang, H. et al. [16] proposed an improved tail light identification model based on YOLOv5s. The CA attention mechanism is introduced in YOLOv5s, EIOU Loss is utilized to solve the category imbalance problem, and Non-Maximum Suppression (NMS) is finally utilized to solve the anchor frame error suppression problem; the mAP value of this model is improved by 9.2% compared with YOLOv5s. Tong et al. [17] proposed a real-time automotive tail light intent detection method that treats tail light detection as a regression question. YOLOv4-tiny is used as the base model, and the detection precision of the model is improved by integrating SPPF and PANet modules. The detection accuracy of the model reaches 86.83%, which is 24.63% higher than the original YOLOv4-minor. Lian et al. [18] proposed a lightweight tail light signal recognition method. The authors designed a lightweight MCA-YOLOv5 network, firstly to make the model lightweight. The YOLOv5s backbone was replaced by MobileNetv3, in addition to the use of the Coordinate Attention module (CA) to improve the model performance.

For vehicle behavior, it includes an intention stage and an execution stage, and the execution stage is directly related to a future section of vehicle trajectory, while the execution stage is governed by driving intention, so both vehicle intention prediction and vehicle trajectory prediction have attracted many scholars. Lee et al. [19] proposed an adaptive cruising controls framework based on Convolutional Neural Network (CNN) lane shift intention inference combined with a prediction controller that converts the real world detected by sensors into a scaled-down version of a birdseye view and feeds it into the CNN framework for the prediction of lane deviation behavior. Li, K. et al. [20] combined the HMM and Bayesian filtering models to recognize the driver’s intention to change lanes. The accuracy of left and right lane change intention recognition reached 93.5% and 90.3%. Huang, L. et al. [21] proposed a deep neural network (DNN)-based approach that takes vehicle transient dynamics as time series data and the sequence, vehicle type, and driver’s expected speed as inputs into the model. The estimated speed vector of the target vehicle in the next time interval is the output of the model. Wehner, C. et al. [22], in order to enhance the interpretability and credibility of Long Short-Term Memory (LSTM) in the field of lane change prediction, the LRP was extended to Layer Normalized LSTM for the real-time prediction and interpretation of lane changes. Liu, L. et al. [23] used a Bayesian parameter optimal support vector machine (SVM) algorithm to solve the multi-parameter and nonlinear problem of the autonomous lane change decision-making process, which exceeded the rule-based model with 86.27% accuracy and was validated using a real vehicle. When the driver is influenced by other vehicles and traffic instructions and forms the driving intention to guide his driving behavior, the vehicle then steps into the implementation phase. Prior to this critical phase, the prediction of the upcoming short-term trajectory of a vehicle is crucial for achieving efficient pre-planning and ensuring driving safety. The core idea is to utilize the vehicle’s past trajectory data, with the help of theoretical analysis or model learning, to reveal and predict the vehicle’s possible travel paths in a particular time period in the future. Park, S.H. et al. [24] used an encoder-decoder architecture to generate the future trajectory sequences of surrounding vehicles in real time, using a long short-term memory (LSTM)-based coder to analyze potential modes in the past trajectories and used an LSTM-based coder to generate future sequences of trajectories. Li, X. et al. [25] proposed the graph-based interactive perceptual trace prediction (GRIP) algorithm, which uses a convolution block to extract features, followed by prediction using an encoder-decoder long-term short-term memory (LSTM) model. The experimental results of a dataset show a 30% improvement in the prediction precision of the model. Su, Z. et al. [26] presented a time-continuous probabilistic trajectory prediction method based on polynomial trajectory parameterization in order to solve problems, such as the existence of errors in the higher order derivatives of acceleration. Luan, Z. et al. [27] proposed an integrated approach for lateral movement prediction by combining driver intention prediction and vehicle behavior recognition. Various driver optimal models were constructed for different drivers, and the probability of driver intention prediction was obtained through game theory. Based on the outcome of driver intention forecasting and vehicular behavior recognition, the driver intention forecasting trajectory and vehicular behavior recognizing trajectory are obtained by using the optimized polynomial trajectory, and the driver intention forecasting and vehicular behavior recognition are inputted into the two Nash optimization functions, respectively, to forecast the combined trajectory.

Although significant progress has been made in the field of vehicle behavior prediction, most prediction models fail to integrate information such as traffic rules and environmental conditions, and the prediction process is complex and opaque. To address the above limitations, this paper proposes a forward vehicle behavior prediction method that combines deep learning techniques with ontological reasoning by introducing the method of ontological reasoning. It uses deep learning techniques to obtain information about the brake lights of the front vehicle, ontology models to integrate multiple factors affecting the behavior of the vehicle, and ontological reasoning to obtain the future behavior of the vehicle. Compared with traditional methods, this method not only utilizes the advantages of deep learning parties in target detection to achieve accurate detection of vehicle brake light information, but it also skillfully integrates diverse external knowledge, such as traffic rules and real-time road conditions, by incorporating ontology. This not only ensures the legal compliance and logical rationality of the prediction results but also enables a flexible response to the changing traffic environment. The application of ontological reasoning makes the prediction process more transparent and easier to understand. The interpretability and reliability of the prediction results are improved.

## 3. Materials and Methods

This paper proposes a front vehicle behavior prediction method that integrates deep learning and ontological reasoning, aiming to achieve accurate prediction of front vehicle behavior by combining advanced computer vision techniques and knowledge representation methods. The method is mainly composed of two core parts: the deep learning part, which is used to detect and recognize the state of the tail light of the front vehicle, and the ontological reasoning part, which is used to construct and parse the logic of the vehicle’s behavior and then predict the future movement of the vehicle.

**Figure 1:**
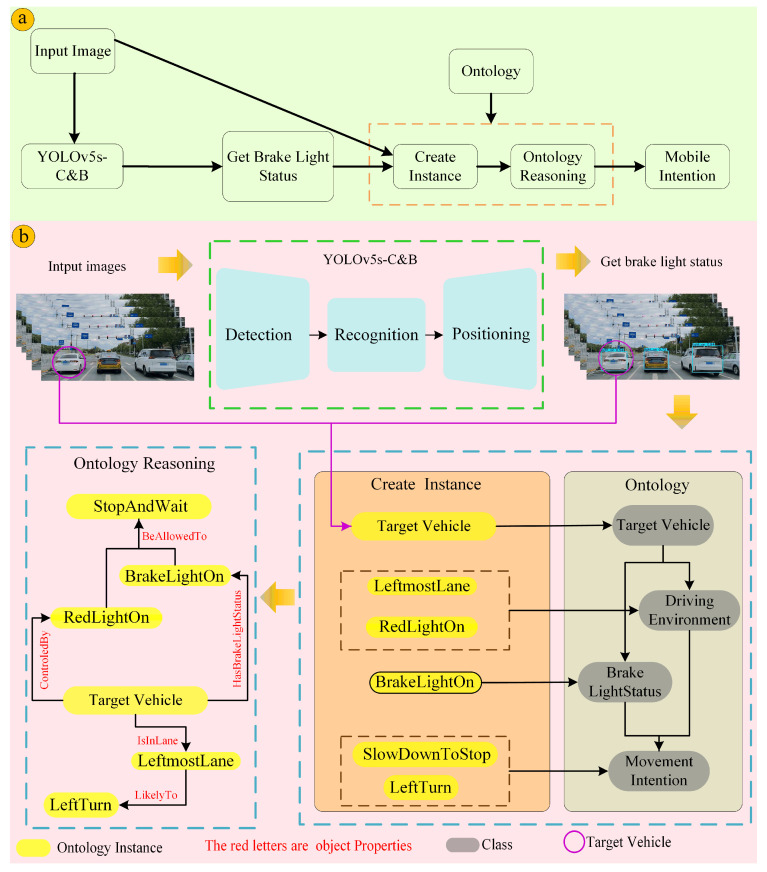
Proposed workflow. (**a**) General diagram of the workflow as detailed in (**b**). The workflow consists of two modules: a brake light detection module based on YOLOv5s-C&B and an ontology reasoning module. Firstly, the vehicle brake light state is detected, combined with the vehicle driving environment, the inference unit is generated, and the ontology inference is finally performed.

As shown in Figure 1, the improved YOLOv5s model is first utilized to detect and accurately classify the brake light status (on or off) of the front vehicle. This step is performed by training the model with a large amount of labeled data so that it can robustly identify changes in tail light status under different lighting and weather conditions, providing a basis for subsequent reasoning regarding vehicle behavior. Subsequently, an ontological reasoning mechanism is introduced to construct an ontological model containing knowledge of vehicle behavior rules, traffic rules, and the road environment. The ontology model not only defines the possible behaviors of the vehicle (e.g., acceleration, deceleration, steering) and their preconditions (e.g., tail light status, driving lane, driving environment, etc.), but also associates the relationship between these behaviors and external environmental factors. Through the detected vehicle brake light information, combined with the vehicle’s driving environment, traffic rules, and traffic signs, etc., the knowledge in the ontology model is used to analyze the possible next behaviors of the vehicle in front of it by applying the rule engine reasoning.

## 4. Deep-Learning-Based Daytime Vehicle Tail Light Detection

**Figure 2:**
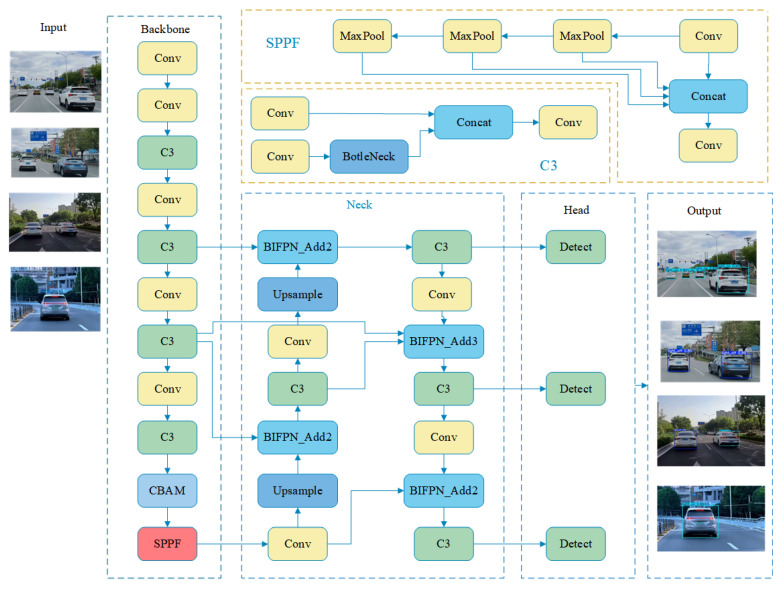
Improved YOLOv5s network structure diagram.

As an intuitive way of inter-vehicle communication, lamp language information contains rich driving intention and state information, which provides new possibilities for predicting the behavior of the vehicle in front. However, the traditional image-processing-dependent light language detection method is susceptible to light and weather changes, requires the frequent adjustment of parameters (e.g., color thresholds), and has poor generalization, making it difficult to adapt to the differences in the tail lights of different vehicles. This limits its application in complex road environments. In recent years, rapidly advancing deep learning technology has provided new ideas for the automatic detection and recognition of lamp language information. The improved network structure is shown in Figure 2.

The YOLO series has become the most popular target recognition method in real-world applications due to its excellent recognition precision and speed. Considering the real-time requirement of the application, we chose YOLOv5s as the base model. YOLOv5s is the smallest model in the YOLOv5 series, which adopts the CSPDarknet structure as the Backbone and combines the FPN and PANet as the Neck part. The detection head of YOLOv5s consists of three different scales of the detection layers, each of which predicts the category and location information of the object. This design enables YOLOv5s to effectively detect objects of different sizes at multiple scales. Despite showing excellent performance, without any improvement, YOLOv5s may not achieve the desired detection accuracy in brake light detection. To address this problem, this paper proposes two improvements to the YOLOv5s model. (1) In an effort to strengthen the feature extraction and detection capabilities of the model on the recognition of complex scenes with small targets, the Convolutional Block Attention Module (CBAM) is introduced in the backbone network. (2) In order to balance the feature information of different scales and improve the detection performance of multi-scale targets, this study optimizes the neck structure in the original network and introduces the weighted Bi-directional Feature Pyramid Network (BIFPN). By using this structure based on the feature pyramid network, the feature information of multiple layers can be better utilized to detect targets of different sizes and scales more accurately.

### 4.1. Introduction of the CBAM Attention Module

When performing brake light detection, it is often necessary to deal with images under different lighting conditions, different viewing angles, and different occlusion situations. Embedding the Convolutional Block Attention Module (CBAM) in the network of YOLOv5s can effectively improve the network performance by increasing the weight of the brake light information, which enables the model to pay more attention to the target-related regions in the image and at the same time suppresses the other useless information to reduce the brake light from being occluded by other objects or interfered with by other light sources. The Convolutional Block Attention Module (CBAM) is composed of two sub-modules, the channel attention module and the spatial attention module, and the whole process of the attention mechanism can be summarized in Equations (1) and (2).

F′ = M_c_(F) ⊗ F (1)

**Figure 3:**
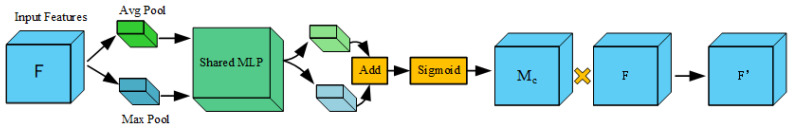
Flowchart of the channel attention module.

**Figure 4:**
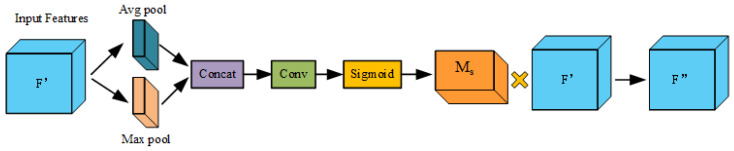
Flowchart of the spatial attention module.

F″ = M_s_(F′) ⊗ F′ (2)

where F is the input feature map, M_c_ is the channel attention map, M_s_ is the spatial attention map, “⊗” stands for element-by-element multiplication, F′ is the result of element-by-element multiplication of the one-dimensional channel attention map and the input feature map, and F″ is the final refined output of the Convolutional Block Attention Module (CBAM). Figure 3 and Figure 4 depict the computation process for each attention graph. The following describes the details of each attention module.

Figure 3 illustrates the workflow of the channel attention module. The input feature graph F (H × W × C) is first aggregated with spatial information through average pooling and maximum pooling operations. The average pooling feature $F_{avg}^{c}$ and the maximum pooling feature $F_{max}^{c}$ are generated. Next, the results of the average pooling and maximum pooling are processed using the shared network. Following this, the outputs of the shared network are aggregated through summation and subsequently passed through a Sigmoid activation, yielding the channel attention map M_c_. This attention map is then utilized to recalibrate the input feature map channel-wise by means of element-wise multiplication, resulting in the enhanced feature map that serves as input to the subsequent spatial attention module. The channel attention module can be summarized as Equation (3).

(3) $$M_{c}(F)=\sigma (\mathrm{MLP}(\mathrm{AvgPool}(F))+\mathrm{MLP}(\mathrm{MaxPool}(F)))\;=\sigma (W_{1}(W_{0}(F_{avg}^{c}))+W_{1}(W_{0}(F_{max}^{c}))).$$

where σ denotes the Sigmoid activation function, W_0_, W_1_ are the MLP operation weights, and $F_{avg}^{c}$, $F_{max}^{c}$ are the average and maximum pooling results of the channel attention module, respectively.

Figure 4 illustrates the workflow of the spatial attention module. The spatial attention module takes the feature map F′ output from the above channel attention module as the input feature map of this module. The input feature maps are firstly pooled using average pooling and maximum pooling operations to obtain the average pooled feature $F_{avg}^{s}$ and the maximum pooled feature $F_{max}^{s}$, which are then concatenated along the channel dimensions and sent to the convolutional layer and generate the spatial weight map M_s_ after passing through the sigmoid function, which is multiplied by F′ to obtain a feature map F″ of the same size as the input feature map and containing both channel and spatial attention. The spatial attention module can be summarized as Equation (4).

(4) $$M_{s}(F)=\sigma (f^{7\times 7}([\mathrm{AvgPool}(F);\mathrm{MaxPool}(F)]))\;=\sigma (f^{7\times 7}[F_{avg}^{s};F_{max}^{s}]).$$

where f^7×7^ denotes the 7 × 7 convolutional kernel, and $F_{avg}^{s}$, $F_{max}^{s}$ are the average pooling and maximum pooling results of the spatial attention module, respectively.

### 4.2. Introduction of the BIFPN Network

Unlike traditional Feature Pyramid Networks (FPN), the weighted Bi-directional Feature Pyramid Network (BIFPN) introduces bidirectional connectivity between neighboring levels of the feature pyramid. This bidirectional connectivity allows information to flow both bottom-up and top-down in the pyramid. Specifically, information can flow from higher-level features to lower-level features or from lower-level features to higher-level features. The weighted Bi-directional Feature Pyramid Network (BIFPN) integrates features at different scales through a feature fusion process, resulting in a more detailed and enriched feature map. The feature fusion process consists of up-sampling and down-sampling operations to adjust the scale of the feature maps, and feature fusion techniques are used to fuse feature maps of different scales. This bi-directional feature fusion helps to effectively capture multi-scale features and improve the accuracy of target detection. In addition, the weighted Bi-directional Feature Pyramid Network (BIFPN) uses a weighted feature fusion mechanism to combine features at different levels. In weighted feature fusion, each input feature map is assigned a learnable weight that reflects the importance of different feature maps for the final detection result. Through the learning process of the network, these weights are automatically adjusted to optimize the performance of target detection.

## 5. Ontology Modeling and Semantic Rule Writing

**Figure 5:**
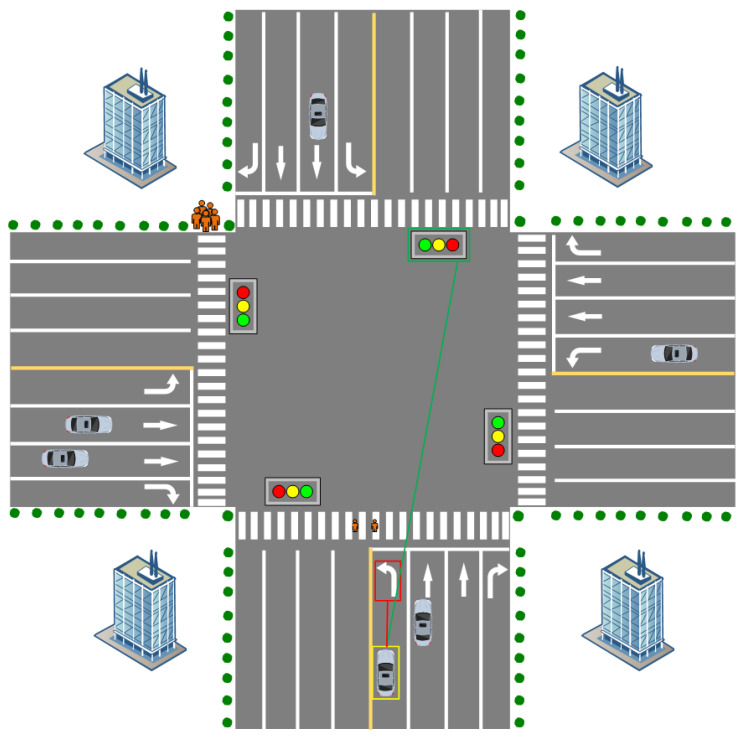
Application Scenario Layout Diagram. The yellow box is the target vehicle, the red box line indicates the road sign’s role in indicating the vehicle, and the green box line represents the traffic light’s control of the vehicle’s behavior.

An ontology model is a conceptualized model for describing domain knowledge that provides an architecture for knowledge representation and reasoning by explicitly defining and classifying entities, attributes, and relationships in the domain. In this paper, the constructed ontology model is used to describe the various entities, attributes, and relationships among them related to vehicle maneuvering intentions, so as to achieve the effective management and utilization of traffic information and vehicle information. Given the limited vehicle information extracted by the visual detection model, we focus on a specific and challenging application scenario, four-lane intersections, where we collect information from vehicles that are entering the intersection to predict their behavior once they enter the intersection. Furthermore, we create an ontology model with this goal. The application scenario layout is shown in Figure 5.

### 5.1. Defining Classes and Class Hierarchies

In constructing the ontology model for predicting the behavior of the front vehicle, we considered a variety of factors that would have an impact on the behavior of vehicles entering the intersection in the application scenarios described in this paper. First, the vehicle type and its size difference is one of the important factors affecting the prediction of front vehicle behavior. Given the differences in the space required by different types and sizes of vehicles when traveling, the model specifically considers this variable; second, as a key signal to guide vehicles to pass in an orderly manner, the different states of traffic signals have a direct impact on vehicle travel decisions. The integration of traffic signal states into the ontology model can improve the accuracy of prediction; furthermore, the instructive role of road signs and markings cannot be ignored as they are not only important references for drivers to navigate but also regulate the behavior of the vehicle, and their inclusion provides important environmental information for the prediction model; finally, as an important medium for the communication between vehicles, the information of the vehicle light language can reflect the driving intention and immediate state of the vehicle intuitively; we constructed the model with this variable in mind. Finally, as an important medium of communication between vehicles, the vehicle’s light language information can intuitively reflect the vehicle’s driving intention and immediate state. When we constructed the ontology model, we especially emphasized the capture of light language information to realize the advanced prediction of the upcoming turns and decelerations of the vehicle in front of us. By comprehensively considering these elements, we aim to build an ontology model that can accurately reflect the actual driving environment of a four-lane intersection and has profound explanatory power for vehicle behavior.

**Figure 6:**
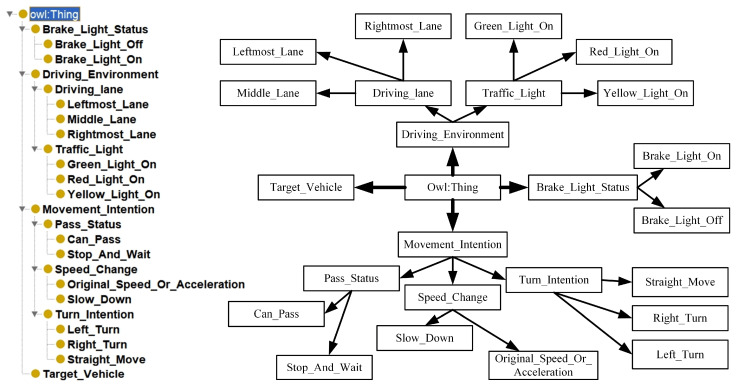
Visual illustration of hierarchy of front vehicle behavior prediction ontology model.

Classes represent different types of entities in a driving scenario. First, we identify and define the core classes that are closely related to the prediction of front-end behavior, and the ontology of front-end behavior prediction consists of four top-level concepts: the target vehicle (Target_Vehicle), the driving environment (Driving_Environment), the state of the brake light (Brake_Light_Status), and the intention to maneuver (Movement_Intention). These categories represent the subject, the external environment, the immediate state, and the future intention of the ex-vehicle’s behavior, respectively. From these four categories of top-level concepts, their substructures are subdivided. One of them, Driving Environment, is a composite category that describes the driving conditions and environment in which the target vehicle is located. It consists of two subcategories: vehicle driving lanes (Driving_lane) and traffic light status (Traffic_Light). The driving lane is used to describe the lane in which the vehicle is currently located, and it has subcategories: the leftmost lane (Leftmost_Lane), the rightmost lane (Rightmost_Lane), and the middle lane (Middle_Lane). The traffic light status is used to describe the current traffic light status, which has subcategories: red light on, green light on, and yellow light on. Traffic light status is used to describe the current traffic light status, which has subcategories: red light on (Red_Light_On), green light on (Green_Light_On), and yellow light on (Yellow_Light_On). The target vehicle is associated with its driving lane and traffic light status through object properties. Maneuvering Intention is used to indicate the future driving intention and plan of the target vehicle. It consists of three subcategories: turning intention (Turn_Intention), speed change (Speed_Change), and whether vehicles can pass (Pass_Status). Turning Intent is used to describe the vehicle’s turning plan, which has subcategories: left turn (Left_Turn), right turn (Right_Turn), and straight ahead (Straight_Move). Speed change is used to describe a vehicle’s speed change plan, which has subcategories: slow down (Slow_Down) and maintain speed or accelerate (Original_Speed_Or_Acceleration). Whether a vehicle is passing or not means that the vehicle needs to judge whether it can pass or needs to stop and wait according to the traffic light signal. It has two subcategories: can pass (Can_Pass) and stop and wait (Stop_And_Wait). The target vehicle is associated with its turning intent and speed change through object attributes. The complete ontology model hierarchy for predicting the behavior of the front vehicle is shown in Figure 6.

### 5.2. Defining Relevant Properties

Next, we established associations between these categories. For example, “Target_Vehicle” is associated with “Driving_Environment”, which indicates the specific driving conditions in which the vehicle operates. The association between “Target_Vehicle” and “Brake_Light_Status” reflects the vehicle’s immediate intention to slow down or stop. The association between “Target_Vehicle” and “Movement_Intention” reveals the vehicle’s future driving direction and speed changes. These associations together constitute a complete description of the behavior of the front vehicle. The complete object properties are shown in Table 1.

Data properties are mainly used to describe the association between classes and data. For a target vehicle, its data properties can include the vehicle number, vehicle size, etc. However, due to the limited vehicle information acquired by the visual detection model, it is not possible to accurately represent the motion characteristics of the target vehicle, such as speed and acceleration, through data properties. Therefore, in this paper, we only set two data attributes, vehicle ID and vehicle type.

### 5.3. Semantic Rule Writing

Rules of inference are based on common sense and general driving behavior. These rules will help us infer the future behavior of the vehicle in front of us based on known information.

The state of the brake lights clearly indicates whether a vehicle is undergoing a deceleration maneuver or not, while the state of the traffic signals directly determines whether a vehicle needs to stop and wait or get permission to pass at an intersection. In a typical four-lane intersection layout, the left lane is designed as a left-turn-only lane, and vehicles traveling in this lane are usually required to perform a left-turn maneuver while obeying the traffic signal. The center lane is used as a straight ahead lane for vehicles traveling straight ahead. As for the rightmost lane, it is a right-turn-only lane. It is particularly noteworthy that, in most cases, when the red light is on, vehicles in this lane can turn right without obstructing vehicular or pedestrian traffic. The partial inference rules developed in this paper for predicting the behavior of the vehicle ahead are shown in Table 2.

## 6. Experimentation and Validation

### 6.1. Dataset Production and Model Training

Since there is no publicly available dataset for vehicle brake light detection, for the detection task described in this paper, we collected dozens of videos captured by a vehicle recorder regarding different roads, traffic conditions, weather conditions, and lighting conditions. These videos not only covered a wide range of driving scenarios but also ensured the diversity and representativeness of the data. Subsequently, we converted these videos into frame sequences, resulting in a total of 5000 images, and carefully labeled the dataset using the Labelimg tool. In order to train and evaluate the model scientifically and efficiently, we divided the dataset into training, validation, and testing sets at a ratio of 8:1:1.

The experiments were conducted using the PyTorch deep learning framework. The Python version was 3.8. The computer configuration CPU was 13th Gen Intel^®^ Core^TM^ i9-13900KF, the graphics card was NVIDIA GeForce RTX4080, the operating system was Windows 11, and CUDA was version 12.2. The initial learning efficiency of the weights used for network training was 0.001, the decay coefficient was 0.0005, the batch size was 8, and the number of training sessions was 500.

**Figure 7:**
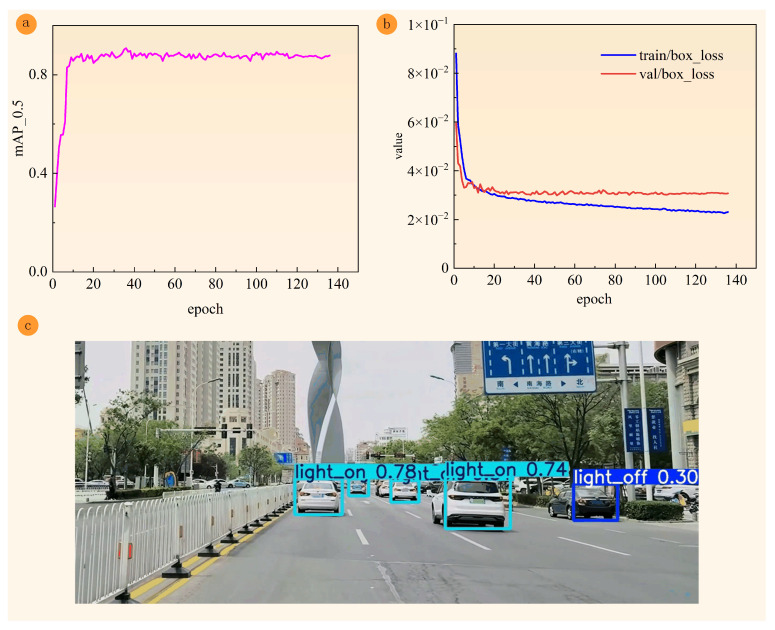
Performance of YOLOv5s-C&B. (**a**) Change curve of mAP@0.5 (**b**) Change curve of bounding box loss on training and test sets (**c**) Detection effect graph of real scene.

Figure 7a demonstrates the trend of mAP@0.5 growth with the rounds of training rounds during the training process of this model. During the first 20 rounds of training, the mAP@0.5 value climbs rapidly from a low starting point, indicating that the model is primed to recognize and localize target objects. As the training process progresses, the growth rate of the mAP@0.5 value gradually slows down and peaks at about the 40th round. At this stage, the improvement in model performance stabilizes, indicating that the model has fully learned the effective information in the training data. In the late stage of training, the mAP@0.5 value may be affected by minor fluctuations due to factors such as random noise, but overall it remains at a high level and stabilizes. Therefore, it can be considered that the model has completed the training process and reached a stable state. As shown in Figure 7b, the bounding box loss on both the training and validation sets gradually decreases with training and eventually stabilizes. This indicates that the model’s localization performance on both training and validation data is improving and there is no significant overfitting or underfitting. Figure 7c shows the detection results of the model in a real scene. From the figure, it can be seen that the model detected the state of the vehicle’s brake light accurately, both on and off, which can be correctly recognized and labeled by the model. This fully proves the effectiveness and accuracy of the model in practical applications.

**Figure 8:**
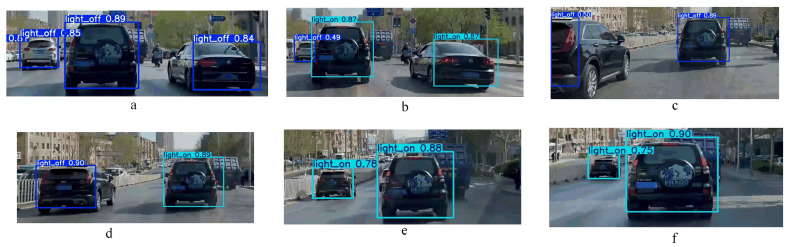
Effect of continuous process detection.

Figure 8 illustrates the brake light detection results for a continuous traveling process. In Figure 8a, only the rightmost vehicle’s brake light is on in the three vehicles, and the middle vehicle’s brake light is also on in Figure 8b. The left vehicle is partially obscured in Figure 8c, and the model still makes the correct detection. And the brake light status of the vehicles in Figure 8d–f can be detected stably all the time in the subsequent process.

**Figure 9:**
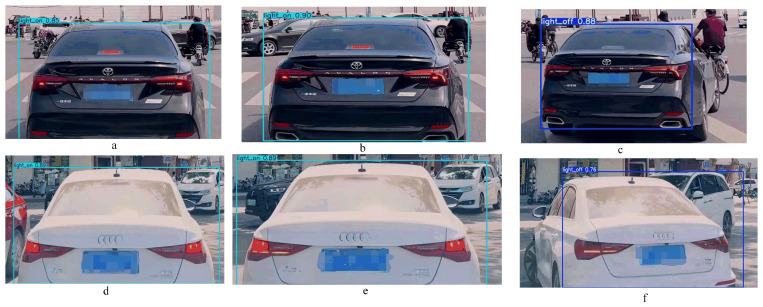
Detection result of the case with steering signal. (**a**,**d**) Brake light activated only. (**b**,**e**) Brake light and turn signal operational simultaneously. (**c**,**f**) Turn signal activated only.

Near intersections, braking behavior is often accompanied by turn signals. Considering that turn signals may interfere with the detection of brake lights, we selected a few cases containing turn signals for our experiments. Specifically, the models correctly detected brake lights when they were either solely on (Figure 9a,d), on concurrently with turn signals (Figure 9b,e), or when only turn signals were illuminated (Figure 9c,f), confirming that turn signals did not interfere with brake light detection.

### 6.2. Ablation Experiments

For the purpose of fully verifying the effectiveness of the proposed algorithm, this study conducted a number of module ablation experiments on the above dataset, which were designed to extensively explore the influence of the Convolutional Block Attention Module (CBAM) and the weighted Bi-directional Feature Pyramid Network (BIFPN) on the algorithm’s performances. A total of four sets of ablation experiments were designed in this study, and the experimental results are shown in Table 3.

The data presented in Table 3 provide a clear understanding of the key experimental results of the improved algorithms in this study. The first set of experiments chose the Yolov5s algorithm as the original model with which subsequent experiments were compared and analyzed; the mAP@0.5 of the original model was 88.3%. These data provide a benchmark as well as a clearer direction for subsequent improvement and optimization. The second set of experiments introduced the Convolutional Block Attention Module (CBAM) on the basis of the original network, and this modification helped to enhance the model’s attention to key information, which can more comprehensively capture the key features in the image, and improved the mAP@0.5 index by 0.8%. The third set of experiments introduced the weighted Bi-directional Feature Pyramid Network (BIFPN) on the basis of the original network, and the addition of this module can solve the problem of multi-scale target detection in tail light detection; the mAP@0.5 index was improved by 0.7% compared with the original network. The fourth set of experimental original networks introduced both the Convolutional Block Attention Module (CBAM) and the weighted Bi-directional Feature Pyramid Network (BIFPN); the mAP@0.5 index was improved by 2.5% compared with the original network, showing significant performance improvement. This result validates the effectiveness of the Convolutional Block Attention Module (CBAM) and the BIFPN network module in enhancing the model detection capability.

**Figure 10:**
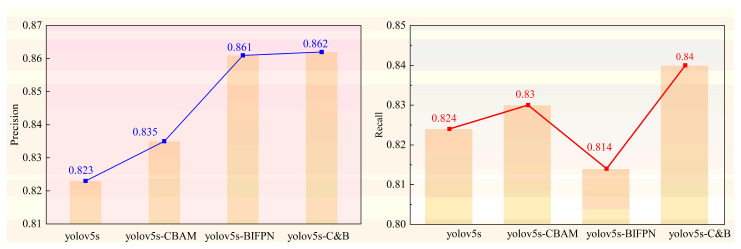
Performance of different models.

Figure 10 illustrates the accuracy and recall of the different models. After the introduction of the Convolutional Block Attention Module (CBAM), the model (YOLOv5s-CBAM) shows a small increase in both precision and recall compared to the original model. The model (YOLOv5s-BIFPN) with the introduction of the weighted Bi-directional Feature Pyramid Network (BIFPN) shows a large degree of increase in precision but a small decrease in recall compared to the original model, and the model (YOLOv5s-C&B) shows a small decrease in performance compared to the original model after the simultaneous entry of the Convolutional Block Attention Module (CBAM) and the weighted Bi-directional Feature Pyramid Network (BIFPN); the model (YOLOv5s-C&B) has a greater degree of growth in precision and recall and outperforms the two single-improved models.

### 6.3. Example Creation of Ontology Model for Predicting Front Vehicle Behavior

**Figure 11:**
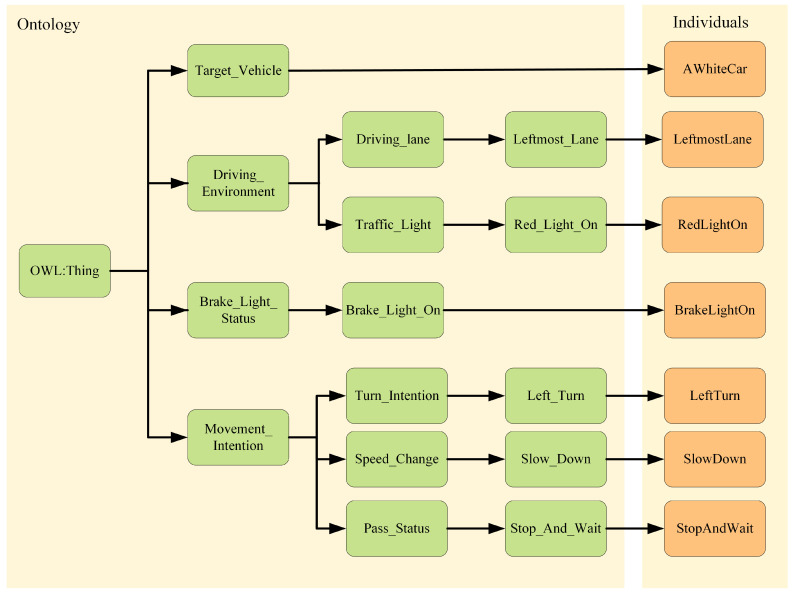
Ontology model instance setup.

In constructing the semantic relationship framework, we refined the scenario shown in Figure 7c into concrete examples. We converted the detected brake light status into concrete instances, namely “BrakeLightOn” and “BrakeLightOff”, which belong to the subclasses of “Brake_Light_Status”. In order to comprehensively and logically link the four top-level concepts mentioned earlier—“Brake_Light_Status”, “Target_Vehicle”, “Driving_Environment” and “Movement_Intention”, we also created related instances under these categories. The instance “AWhiteCar” belongs to the “Target_Vehicle” class. The instances “LeftmostLane” and “RedLightOn” belong to the “Driving_Environment” class. The instances “LeftTurn”, “SlowDown”, and “StopAndWait” belong to the “Movement_Intention” class. The schematic diagram of instance creation is shown in Figure 11. At the same time, according to the different characteristics of the different conceptual classes to which the instances belong, the object and data attributes corresponding to the instances are added. After that, the specific instance rules are converted into SWRL rules.

### 6.4. Predictive Reasoning Results for Front Vehicle Behavior

After creating the corresponding instances in the ontology model for predicting the behavior of the front vehicle, we performed inference experiments in the protege software. First, the brake light status, driving lane, and traffic light of the target vehicle are converted into individual instance knowledge, which is stored in the ontology model and represented as Drools facts. Then, the multiple SWRL rules constructed in the previous section are converted into Drools rules, and finally the Drools inference engine is run to perform inference based on the facts and rules.

**Figure 12:**
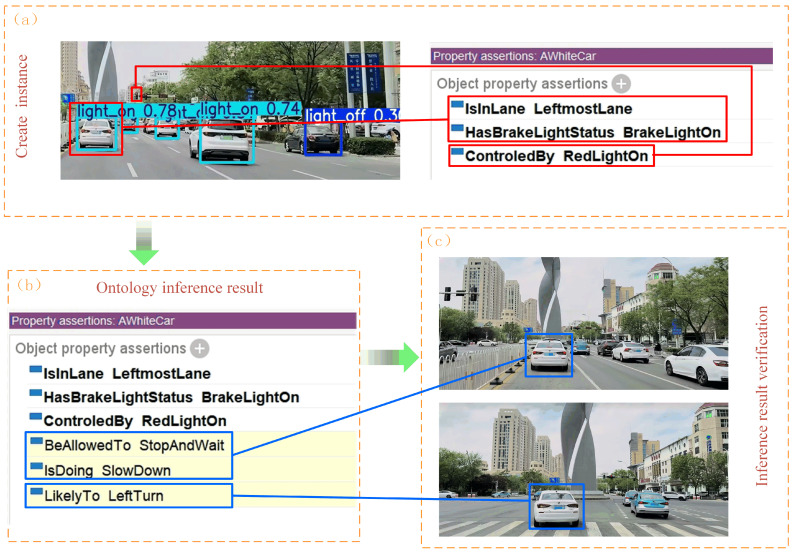
Ontology reasoning results. (**a**) The information utilized for the reasoning and the source of that information. (**b**) Yellow underlining shows the model’s reasoning results. (**c**) The subsequent behavior of the vehicle. The blue boxed line connects the inference result and its corresponding vehicle behavior.

As can be seen from the inference results demonstrated in Figure 12, the front vehicle behavior prediction ontology model constructed in this paper exhibits reliable performance and is able to accurately predict the future behavioral dynamics of the target vehicle. As shown in Figure 12b, in the object properties list of “AWhiteCar” (i.e., the target vehicle), there is a new key property, “IsDoing SlowDown”, which is based on the target vehicle’s brake light illumination to deduce that the target vehicle is performing deceleration behavior. Meanwhile, “BeAllowedTo StopAndWait” is the result of the model’s speculation from the information that the red light is on, i.e., the target vehicle needs to stop and wait for the passing signal after entering the intersection. In addition, the labeling of the “Likelyto LeftTurn” property indicates that the target vehicle has a high probability of performing a left-turn maneuver after receiving a passing signal. Therefore, the model’s prediction of the target vehicle’s behavior can be summarized as follows: the target vehicle is decelerating, after entering the intersection, the target vehicle needs to stop and wait for a signal that it can pass (the green light is on), and the target vehicle will make a left turn after leaving the intersection. Figure 12c shows the actual behavior of the target vehicle, and the prediction result of the ontology model corresponds exactly to it, verifying the accuracy of the prediction made by the ontology model. This example validation shows that the front vehicle behavior prediction model constructed in this paper is able to predict the behavior of the vehicle based on the acquired vehicle information.

## 7. Conclusions

In order to compensate for the inability of deep learning techniques to integrate traffic rules and environmental information limitations and to improve the interpretability and reliability of prediction results, in this paper, we introduce the method of ontological reasoning and propose a method for predicting the behavior of the vehicle ahead that combines deep learning and ontological reasoning. The method uses deep learning technology to obtain the brake light information of the vehicle in front, uses the ontology model to integrate multiple factors affecting vehicle behavior, and uses ontology reasoning to predict the future behavior of the vehicle. In order to achieve accurate detection of brake lights, we introduced the Convolutional Block Attention Module (CBAM) and the weighted Bi-directional Feature Pyramid Network (BIFPN) in the YOLOv5s model, which improved the model’s precision and mAP@0.5 by 3.9% and 2.5%, respectively. After that, we selected a four-lane intersection as an application scenario, analyzed multiple factors affecting vehicle behavior, and created an ontology model for predicting the behavior of the front vehicle. In the final inference experiment, the ontology model created in this paper accurately and completely reasoned out the future behavior of the target vehicle, which verified the reasonableness and practicability of the front vehicle behavior prediction method constructed in this paper.

In this paper, we successfully integrated traffic rules and environmental information by introducing ontology, which effectively enhanced the accuracy and interpretability of prediction results. Of course, as the requirements of self-driving cars increase, the vehicle behavior prediction module should identify all possible future movements so that the car can work reliably. This requires taking into account the movement trends of surrounding vehicles, road conditions, traffic rules, and even weather factors. Then, a single sensor will not be able to satisfy this need; multi-sensor fusion for vehicle behavior prediction is the direction of our future work. The addition of LIDAR, millimeter wave radar, and GPS sensors will provide more abundant data support for the vehicle behavior prediction module.

## Figures and Tables

**Table 1:** Object property settings.

Name of Object Property	Domain	Range
IsInLane	Target_Vehicle	Driving_Lane
IsDoing	Target_Vehicle	Speed_Change
ControledBy	Target_Vehicle	Traffic_Light
HasBrakeLightStatus	Target_Vehicle	Brake_Light_Status
LikelyTo	Target_Vehicle	Turn_Intention
BeAllowedTo	Target_Vehicle	Pass_Status

**Table 2:** Ontological rules for the future behavior of the front car.

No	Inference Rules
Rule 1	If the target vehicle’s brake lights are on, then the vehicle is slowing down.
Rule 2	If the target vehicle’s brake lights are not illuminated, then the vehicle is accelerating or traveling at the same speed.
Rule 3	If the target vehicle is traveling in the leftmost lane, then the vehicle will turn left.
Rule 4	If the target vehicle is in the rightmost lane, then the vehicle will turn right.
Rule 5	If the target vehicle is traveling in the center lane, then the vehicle will go straight.
Rule 6	If the target vehicle’s brake lights are illuminated and the red light is illuminated, then the vehicle is slowing down and has to stop and wait.
Rule 7	If the target vehicle’s brake light is illuminated and the green light is illuminated, then the vehicle will slow down.
Rule 8	If the target vehicle’s brake light is illuminated and the yellow light is illuminated, then the vehicle is slowing down and has to stop and wait.

**Table 3:** Summary of ablation experiment results. (“√” indicates that this module is added).

Group	CBAM	BIFPN	mAP@0.5
1			0.883
2	√		0.891
3		√	0.89
4	√	√	0.908

## Data Availability

The raw data supporting the conclusions of this article will be made available by the authors on request.
